# Risk of Dementia Associated with Elevated Plasma Homocysteine in a Latin American Population

**DOI:** 10.4061/2009/632489

**Published:** 2009-08-23

**Authors:** Inara J. Chacón, Aldrín E. Molero, Gloria Pino-Ramírez, José A. Luchsinger, Joseph H. Lee, Gladys E. Maestre

**Affiliations:** ^1^Gertrude H. Sergievsky Center, Columbia University, New York, NY, USA; ^2^Unit of Medical Genetics, University of Zulia, Maracaibo, Venezuela; ^3^Neurosciences Laboratory, Institute for Biological Research, University of Zulia, Maracaibo, Venezuela; ^4^Department of Neurology, Albert Einstein College of Medicine, Bronx, New York, NY 10461, USA; ^5^Center for Psychological Research, Universidad Rafael Urdaneta, Maracaibo, Venezuela; ^6^Taub Institute for Research of Alzheimer's Disease and the Aging Brain, Columbia University, New York, NY, USA; ^7^Division of General Medicine, Department of Medicine, Columbia University College of Physicians and Surgeons, New York, NY, USA; ^8^Department of Epidemiology, School of Public Health, Columbia University, New York, NY, USA

## Abstract

The relationship between total homocysteine (tHcy) and dementia risk remains controversial, as the association varies among populations and dementia subtypes. We studied a Venezuelan population that has high prevalence of both elevated tHcy and dementia. We tested the hypotheses that (1) elevated tHcy is associated with increased dementia risk, (2) the risk is greater for vascular dementia (VaD) than for Alzheimer's disease (AD), and (3) a history of stroke may partly explain this association. 2100 participants (≥55 years old) of the Maracaibo Aging Study underwent standardized neurological, neuropsychiatric, and cardiovascular assessments. Elevated tHcy was significantly associated with dementia, primarily VaD. When history of stroke and other confounding factors were taken into account, elevated tHcy remained a significant risk factor in older (>66 years), but not in younger (55–66 years) subjects. Ongoing studies of this population may provide insight into the mechanism by which tHcy increases risk for dementia.

## 1. Introduction

Hyperhomocysteinemia has been linked to a number of pathological changes in the endothelium and small vessels [1–6]. Oxidized metabolites of homocysteine have been associated with intraneuronal accumulation of amyloid-*β* (A*β*) 42 and neurotoxicity [7, 8]. These changes have been proposed as the underlying mechanism explaining epidemiological observations that moderate elevation of total plasma homocysteine (tHcy) is associated with an increased risk of cardiovascular and cerebrovascular disease [9] and dementia [10, 11]. However, the relationship between tHcy and dementia is complex, because tHcy levels vary with sex and age [12–14] and are influenced by diet (including folate intake), lifestyle, health status, history of medication, and genetic factors [12, 15–18]. Risk of dementia increases with age and is influenced by many of the same factors [19]. The interaction of these multiple variables makes it difficult to discriminate the independent contribution of tHcy to dementia risk. Moreover, because most previous studies examined subjects who were 65 years of age or older [10, 20–22], those studies lacked a sufficiently broad age range necessary to distinguish the effects of age from that of elevated tHcy. In addition, most research examining tHcy as a risk factor for dementia has been carried out in developed countries [10, 23–27]; few studies have addressed this issue in populations of Hispanic descent residing in the developing world, particularly in Latin America, where dietary supplementation with folate and multivitamins is uncommon [28, 29]. 

In the current study, we examined the association between tHcy level and risk of dementia in an elderly population residing in the Caribbean city of Maracaibo, Venezuela. This population offers an opportunity to evaluate the association between tHcy level and dementia risk, because it has a high prevalence of dementia [30], mean tHcy levels that are substantially higher than those in the U.S. or Europe [14], and limited dietary and medical interventions to prevent or treat hyperhomocysteinemia [28, 31]. In addition, the study participants included individuals 55 years of age and older, providing information about tHcy as a risk factor for dementia in an age range that has not been well studied. Specifically, the present study tested the hypotheses that ( 1) high tHcy is related to increased risk of dementia; ( 2) the risk is greater for vascular dementia (VaD) than for Alzheimer's disease (AD), and ( 3) a history of stroke, representing a major cerebrovascular disease event, may be part of the pathway explaining this association. 

## 2. Materials and Methods

### 2.1. Participants and Setting

Subjects were participants in the Maracaibo Aging Study (MAS), a population-based cohort study that is investigating age-related diseases among 2437 elderly residents of the community of Santa Lucia, Maracaibo, Venezuela [32]. The present study includes only baseline cross-sectional data for the 2100 subjects that had tHcy measurements. Sex and age distributions, educational level, frequency of traditional cardiovascular risk factors (CVRFs), and prevalence of dementia did not differ significantly between the evaluated group and the 337 subjects without tHcy measurements (data not shown). Information on hypercholesterolemia, diabetes, smoking status, hypertension, and stroke was not available for analysis in a small percentage of cases, due to the facts that reliable information could not be obtained, that some subjects did not respond to certain questions, or that some assays failed. However, the proportion of individuals with missing values was not significantly different among demented or nondemented participants. The University of Zulia Institutional Ethical Review Board approved the MAS, and informed consent was obtained from all participants.

### 2.2. Clinical Evaluation

The clinical evaluation included a full battery of neuropsychiatric examinations that integrated medical and family history with history of risk factors for dementia. Standardized cardiovascular evaluation, nutritional assessment, and neuropsychological testing were also performed for every participant. For participants with dementia, information concerning risk factors was obtained from family informants. Methods are described in detail elsewhere [30]. A diagnosis of dementia was determined by consensus in a diagnostic conference of physicians, psychologists, and social workers, based on the criteria in the *Diagnostic and Statistical Manual of Mental Disorders* (4th edition) [33]. AD was diagnosed following the criteria of the NINCDS/ADRDA [34]. The criteria of the State of California Alzheimer's Disease Diagnostic and Treatment Centers (ADDTC) [35] were used for diagnosis of VaD. The ADDTC criteria require evidence of two or more ischemic strokes (at least one infarct outside the cerebellum) by history, neurological signs, and/or neuroimaging studies (CT or T1-weighted MRI), or a single stroke with an evident temporal relationship between the stroke and onset of dementia. In the absence of a defined stroke, clinical and neuroimaging evidence for Binswanger's disease is sufficient to make the diagnosis of possible VaD. History of stroke was taken from self-reports, which defined stroke as an acute neurological deficit lasting more than 24 hours that required medical assistance. 

### 2.3. Laboratory Analyses

Plasma tHcy, vitamin B12, and folate were measured using blood samples obtained between 7 : 00 and 8 : 00 AM, after overnight fasting. All blood samples were obtained and processed at the Cardiovascular Center of the University of Zulia (CCUZ) in Maracaibo. Samples were immediately placed in crushed ice and protected from light and were processed within one hour of collection. The biochemical assays for all samples were performed at the CCUZ. As a quality control measure, a subset of samples (*n* = 40) was tested at the University of Texas Southwestern Medical School, using a method based on that of Araki and Sako [36]. Apolipoprotein E* (APOE) * was genotyped using a modified version [37] of the technique described by Hixson and Vernier [38]. Details of sample collection and analyses have been previously published [14].

### 2.4. Statistical Analysis

Means for demographic characteristics were compared using *t*-tests, and differences in the distribution of cardiovascular risk factors between groups (e.g., demented versus nondemented) were evaluated using the Pearson *χ* ^2^ test. Levels of tHcy, vitamin B12, and folate were log-transformed to fit normal distributions. Odds ratios (ORs) and 95% confidence intervals (CI) were calculated using two logistic regression models. Model 1 adjusted for age, sex, education, diabetes, folate, vitamin B12, and APOE genotype. Model 2 adjusted for a history of stroke, in addition to all of the covariates in Model 1. The latter model was designed to assess whether stroke was an intermediate factor [39], that is, a factor in the pathway in the relationship between tHcy and dementia [5, 9, 40]. Therefore, Model 2 was intended to determine if the association between tHcy and dementia was partly mediated by stroke [41].

We conducted analyses relating tHcy level to the risk of dementia for the entire population, and also for two age groups differentiated using the median age of the entire sample (66 years) as the cut point. This was done to assess the effect of elevated tHcy on the risk of dementia in individuals between 55 and 66 years of age, since this age range is not usually studied. We also added an interaction term to the logistic regression model to evaluate a possible interaction between age group and tHcy. For this purpose, tHcy ≥14 *μ*mol/L was used to define hyperhomocysteinemia [10]. 

Finally, we evaluated tHcy as a risk factor for the principal forms of dementia, AD and VaD. To do this, we used only AD or VaD cases and eliminated individuals suffering from other forms of dementia or from mixed AD and VaD. All statistical analyses were performed using SPSS software, Version 12 (SPSS Inc., Chicago). 

## 3. Results

### 3.1. Population Characteristics

Mean age of participants was 67.2 years (Table 1). The ratio of women to men was 2 : 1 for the entire study population. Men were slightly younger than women and had higher levels of education. Women were more likely to have hypercholesterolemia, and men were more likely to be current smokers. Hypertension, defined as systolic blood pressure ≥140 mm Hg and diastolic pressure ≥90 mm Hg, was highly prevalent in this population, compared to published reports for other Hispanic populations: 84.0% in this study versus 66.4% in the Northern Manhattan Study [21]. The prevalence of self-reported stroke was 5.4%. Frequency of the allele *ε*4 of the Apolipoprotein E gene (ApoE-*ε*4) was 12% (Table 1).

### 3.2. Demented versus Nondemented Individuals

The overall prevalence of dementia was 8% (Table 1). Demented individuals were significantly older than nondemented individuals (77.8 versus 66.3 year, resp.), had fewer years of formal education (3.2 versus 5.8 years), lower folate levels (4.8 versus 5.3 ng/mL), and higher tHcy levels (18.6 versus 13.9 *μ*mol/L, *P* < .05 in all cases) (Table 1). Prevalence of hypertension, current smoking, and hypercholesterolemia did not differ between demented and nondemented individuals; however, the prevalence of diabetes (19.0 versus 12.6%) and of stroke (23.7 versus 3.7%) was significantly higher (*P* < .01 in both cases) in demented individuals than in nondemented individuals. Frequency of the ApoE-*ε*4 allele was significantly higher in demented individuals (16.6 versus 11.1%, *P* < .01). 

### 3.3. Homocysteine, Stroke, and Risk of Dementia

The overall prevalence of hyperhomocysteinemia (tHcy ≥ 14 *μ*mol/L) was high (40.2%) in this cohort (Table 1). Demented individuals were twice as likely as nondemented individuals to have hyperhomocysteinemia (69.4 versus 37.6%, *χ* ^2^ = 64.9, *P* < .0001). 

When all subjects were examined together, each unit increase in log-transformed tHcy level corresponded to a fourfold increase in the risk for dementia after adjustments for age, sex, education, diabetes, folate, vitamin B12, and APOE genotype (Table 2, Model 1). Adjustment for stroke history (Model 2) had little effect on the odds ratio (OR) for the total study population. 

When the sample was stratified into two age groups, the unadjusted ORs for dementia associated with tHcy were similar for individuals 55–66 years and for individuals >66 years (Table 2). When adjusted for age, sex, education, diabetes, APOE genotype, folate, and vitamin B12 (Model 1), the ORs remained significant for both age groups, but the OR for the younger group was slightly higher than that for the older group (6.3 versus 4.2). 

Stroke was strongly associated with dementia in both age groups (unadjusted OR = 11.6, 95% C.I. = 4.5–29.7 for the younger group; OR = 7.3, 95% C.I. = 4.3–12.3 for the older group, *P* < .001), even after adjustment for sex, age, education, diabetes, APOE genotype, folate, vitamin B12, and tHcy (OR = 22.5., 95% C.I. = 6.0–84.4 for the younger group; OR = 7.5, 95% C.I. = 4.0–14.0 for the older group). When adjusted for stroke, the OR for dementia associated with tHcy was relatively constant for individuals >66 years (Table 2, Model 2). However, the OR for the younger group was reduced two folds and was no longer statistically significant, suggesting that stroke is acting as a mediator between elevated tHcy and dementia. 

For the total study population, 74 cases of dementia were clinically classified as AD, and 48 as VaD. After adjusting for covariates, elevated tHcy was a significant risk factor for VaD, but not for AD (Table 3). 

## 4. Discussion

Hyperhomocysteinemia is a significant risk factor for dementia in the Maracaibo Aging Study population. Elevated tHcy was associated with a fourfold increase in dementia risk in both younger and older age groups, and the magnitude of risk was even higher in younger than in older subjects. Thus, the relationship between tHcy and dementia exists over a broad age range. However, our results suggest that a history of stroke mediates the relationship between tHcy and dementia, and that this effect may decline with age. When stroke history was considered (Table 2, Model 2), the magnitude of dementia risk associated with elevated tHcy was reduced in the younger group, but not in the older group. The reduced risk in the younger group suggests that elevated tHcy may influence the risk of dementia by increasing the risk of cerebrovascular disease and stroke-related deficiencies, corroborating previous reports [25, 27, 42]. In other words, stroke may be an intermediate factor [39] in the disease-causing pathway of tHcy. Alternatively, elevated tHcy and stroke may act as independent risk factors, with the influence of stroke overwhelmingly stronger than that of tHcy. To test this hypothesis, we will need to examine the temporality, which cannot be determined from the present cross-sectional study. Our ongoing longitudinal study of the MAS population will test this hypothesis.

In addition to disclosing age-related changes in the relationship between hyperhomocysteinemia and dementia, results of the present study showed that risk for VaD related to tHcy was much higher than risk for AD (Table 3), further supporting the role of cerebral vascular pathways [1, 4–6, 9, 22]. The relationship between hyperhomocysteinemia and AD is controversial [10, 27, 43]. Recent studies have suggested that tHcy is a marker of coexisting vascular conditions, rather than a direct risk factor for AD [44–46]. The overall role of elevated tHcy as a risk factor for stroke and dementia is still unclear. A trial of supplementation of vitamins B6 and B12 and folate showed no apparent benefit to the secondary prevention of stroke [47]. A clinical trial using the same supplements for participants ≥65 years of age with tHcy ≥ 13 *μ*mol/L found lower tHcy levels in the treatment group after two years, but no differences in cognition [48]. However, meta-analysis of clinical trials suggested that longer treatment with folate is associated with improvement in stroke risk [49], and this needs to be explored in relation to dementia. 

One limitation of our study is its cross-sectional nature, restricting our inferences about causality. Longitudinal data from the ongoing MAS will provide further insight into the mechanisms involved in the relationship between hyperhomocysteinemia and dementia. The inclusion of a younger cohort allowed us to examine the influence of tHcy in preclinical stages; however, as expected, the younger cohort had a relatively small number of dementia cases (23 out of 1094 participants), resulting in a large confidence interval. Therefore, the observed differences between age groups should be interpreted with caution. Finally, our clinical definition of stroke did not include data on “silent” cerebrovascular disease from brain imaging, so prevalence of cerebrovascular disease may have been underestimated. 

To the best of our knowledge, the present study provides one of the first assessments of elevated tHcy as a risk factor for dementia for individuals of Hispanic descent living under the nutritional and environmental conditions of a developing country. The only other study in populations of Latin America published to date simply examined the correlation between tHcy level and MMSE value but did not diagnose subjects as demented or differentiate between different types of dementia [29]. The Maracaibo Aging Study is well suited to addressing some of the limitations observed in earlier reports on the relationship between tHcy and dementia. The MAS includes a large subject population of individuals with similar genetic background and lifestyle, and for the most part, unexposed to different forms of interventions and treatments. Thus, the relationship between tHcy and dementia in this population may be less affected by confounding factors. By studying a relatively homogeneous population, some of the inconsistencies of the earlier studies on heterogeneous populations are reduced. Earlier studies included diverse ethnic groups with varying environmental exposures: epidemiologic studies of Caucasian populations in the U.S. and Great Britain [10, 22], and a study of Hispanics residing in North America [24] did find a significant correlation between tHcy levels and dementia, while a study of a multiracial community in Manhattan [21] did not. In some cases, the association was attributed to vitamin deficiencies, rather than to effects of elevated tHcy [20]. In the current study of the MAS population, the association found with dementia was independent of folate and vitamin B12 levels. 

## 5. Conclusions

The present study examined the association between elevated tHcy and dementia over a broader age range than most previous population-based studies. Although arbitrarily selected, the age cut point allowed evaluation of the association of tHcy and dementia in a significant number (1094) of relatively young individuals (55–66 years). Although longitudinal data are needed to assess causality, the demonstrated relationship between tHcy and dementia in the two age groups of this Latin American population provides new insight into the impact of hyperhomocysteinemia throughout life, and into the relevance of tHcy as a risk factor in populations from developing countries, where hyperhomocysteinemia is common. 

## Figures and Tables

**Table 1:** Characteristics of the Maracaibo Aging Study population.

Characteristics	All	Demented	Not demented
Population, no. (% females)	2100 (67.0)	169 (74.0)	1931 (66.4)
Age, mean (SD), y	67.2 (9.0)	77.8 (8.6)**	66.3 (8.3)
Illiteracy, *N* (%)	298/2100 (14.2)	61/169 (36.1)**	237/1931 (12.3)
Education, mean (SD), y	5.6 (4.0)	3.2 (3.2)**	5.8 (4.0)
Hypercholesterolemia, *N* (%)	711/1553 (45.8)	49/121 (40.5)	662/1432 (46.2)
Diabetes, *N* (%)	287/2099 (13.7)	32/169 (19.0)*	255/1930 (13.2)
Current smoker, *N* (%)	350/2100 (16.7)	20/169 (12.0)	329/1931 (17.0 )
Hypertension, *N* (%)	1739/2100 (82.8)	141/169 (83.4)	1598/1931 (82.8)
Stroke, *N* (%)	113/2099 (5.4)	40/169 (23.7)**	72/1930 (3.7)
Hyperhomocysteinemia, *N* (%)	844/2100 (40.2)	117/169 (69.2)*	727/1931 (37.6)
tHcy, mean (SD), mol/L	14.2 (6.3)	18.6 (7.5)*	13.9 (6.0)
Folate, mean (SD), (ng/ml)	5.3 (2.9)	4.8 (3.3)*	5.3 (3.0)
Vit B12, mean (SD), (pmol/ml)	416.9 (335.0)	426.3 (341.5)	415.9 (334.5)
APOE allele frequency			
ApoE-*ε*2	0.046	0.035	0.047
ApoE-*ε*3	0.838	0.769	0.842
ApoE-*ε*4	0.116	0.166*	0.111

*P < .05; **P < .001, between demented and not demented groups.

**Table 2:** Odds ratios (ORs) for risk of dementia associated with log-transformed levels of total homocysteine (tHcy).

		Number	Crude		Number	Model 1^a^		Number	Model 2^b^	
Group	Risk factor	Cases/Total	OR	95% CI	Cases/Total	OR	95% CI	Cases/Total	OR	95% CI

Total sample	log(tHcy)	169/2100	6.3**	4.2–9.3	152/1787	4.2**	2.4–7.3	152/1786	3.6**	2.0–6.4
55–66 years	log(tHcy)	23/1094	4.6*	1.7–12.3	21/927	6.3*	1.6–24.6	21/927	4.5	0.9–20.5
>66 years	log(tHcy)	146/1006	4.7**	2.9–7.5	131/860	4.2**	2.3–7.8	131/859	3.8**	2.0–7.2

^a^Model 1 adjusted for age, sex, education, diabetes, APOE genotype, folate, and vitamin B12; ^b^Model 2 adjusted for age, sex, education, diabetes, APOE genotype, folate, vitamin B12, and stroke, *P ≤ .02; **P < .001.

**Table 3:** Comparison of risk associated with total homocysteine (tHcy) for Alzheimer's disease (AD) and vascular dementia (VaD).

		No.	Crude		No.	Adjusted^a^	
Group	Risk factor	Cases/Total	OR	95% CI	Cases/Total	OR	95% CI

AD	log(tHcy)	74/2005	4.3*	2.5–7.5	65/1701	1.9	0.9–4.2
VaD	log(tHcy)	48/1979	7.8*	4.1–15.1	45/1681	4.6*	2.0–11.1

^a^Adjusted model includes age, sex, education, diabetes, folate, vitamin B12, and APOE genotype; *P ≤ .001, criteria from the NINCDS/ADRDA [34] were used to diagnose AD and criteria from the ADDTC [35] to diagnose VaD.
